# FoCUS and non-invasive hemodynamics monitoring in neonatal sepsis

**DOI:** 10.1007/s00431-025-06283-6

**Published:** 2025-06-30

**Authors:** Noura Abdou, Mohammed Rowisha, Heba Elmahdy, Osama Tolba, Nihal Shihab, Asmaa Elmesiry

**Affiliations:** 1 Pediatrics, Faculty of Medicine, Tanta University, Tanta, Gharbeya Egypt; 2 Neonatology, Faculty of Medicine, Tanta University, Tanta, Gharbeya Egypt; 3 Pediatric Cardiology, Faculty of Medicine, Tanta University, Tanta, Gharbeya Egypt; 4 Public Health, Faculty of Medicine, Tanta University, Tanta, Gharbeya Egypt; 5 Faculty of Medicine, Pediatric Department, Gharbia Governorate, El Bahr StTanta Qism 2, Tanta, 31527 Egypt

**Keywords:** Neonatal sepsis, Electrical cardiometry, Focused cardiac ultrasound

## Abstract

Sepsis is one of the most encountered pathologies in the neonatal intensive care unit (NICU) and is associated with significant morbidity and mortality, especially in preterm neonates. In this study, we aimed to assess early hemodynamic status in preterm neonates diagnosed with sepsis by electrical cardiometry and compare its utility to functional echocardiography in NICU. This is a case–control prospective observational study that enrolled 70 septic preterm neonates (34 0/7 to 36 6/7 weeks gestational age) who were admitted to Tanta University NICU and met the criteria for diagnosis of neonatal sepsis; meanwhile, cases with septic shock were excluded. A non-sepsis group of 70 newborns matched for gestational age and sex was included as a control group. Bedside focused cardiac ultrasound (FoCUS) and electrical cardiometry monitoring were performed on the 2nd day of the clinical diagnosis of sepsis. Stroke volume (SV), cardiac output (CO), and cardiac index (CI) measurements by both echocardiography and electrical cardiometry were significantly higher in the sepsis group compared to the non-sepsis group. Systemic vascular resistance (SVR) and systemic vascular resistance index (SVRI) were significantly lower in the septic group compared to the non-sepsis group. Correlations between electrical cardiometry and focused cardiac ultrasound parameters were significantly positive regarding SV, CO, CI, SVR, and SVRI in all the studied groups.

*Conclusions*: Electrical cardiometry could be considered a useful and promising tool for early assessment of hemodynamics in preterm neonates with sepsis coinciding with echocardiography.

**Table:** 

**What is Known:** • *Preterm infants diagnosed with sepsis can develop cardiovascular instability due to the unique features of their cardiovascular function and reserve*. • *Prompt identification of patients with progression of sepsis severity may be accomplished through frequent clinical assessments and monitoring of the changes in their physiological indices (heart rate, BP, urine output, capillary refill time, and neurologic status) and biochemical variables (blood gas, lactate, electrolytes, and creatinine)*.
**What is New:** • *Electrical cardiometry could be considered a useful and promising tool for the early assessment of hemodynamics in preterm neonates with sepsis coinciding with echocardiography*.

**Supplementary Information:**

The online version contains supplementary material available at 10.1007/s00431-025-06283-6.

## Introduction

Neonatal sepsis is a systemic infection occurring in infants ≤ 28 days of life, and it is an important cause of morbidity and mortality of newborns. Moreover, studies have shown an increased rate of neonatal infection in association with lower gestational age and lower birth weight [1, 2].

The neonatal cardiovascular response to sepsis varies from that perceived in older children and adults and may result in a hyperdynamic or a hypodynamic circulation. Sepsis-induced cardiovascular dysfunction includes myocardial dysfunction and vasoregulatory failure that could lead to systemic vasodilation or vasoconstriction [3].

Functional echocardiography is a non-invasive method for assessing myocardial function and hemodynamic parameters as the cardiac output (CO) in infants. However, it is operator-dependent and requires a specific technique. Electrical cardiometry (EC) is a feasible bedside non-invasive tool that measures thoracic electrical bioimpedance (TEB) and hemodynamic parameters such as stroke volume (SV) and cardiac output (CO), contractility (expressed as contractility index, ICON), systemic vascular resistance (SVR), and thoracic fluid content [4].

This study aimed to assess early hemodynamic status in septic preterm neonates by using electrical cardiometry and comparing its utility to functional echocardiography in the NICU. We hypothesized that electrical cardiometry could facilitate continuous bedside hemodynamic monitoring in septic preterm infants and help in the early management of any instability.

### Methods

This case–control prospective observational study was conducted in the NICU of Tanta University Hospitals after it was approved by the local ethical committee of The Faculty of Medicine, Tanta University (No. 33069/04/19). Written parental consent was obtained prior to cases enrollment. The study included 70 preterm newborns (gestational age ranged from 34 0/7 to 36 6/7 weeks calculated from the first day of the last normal menstrual period and by using the New Ballard score [5]) who met the diagnostic criteria of neonatal sepsis, as defined by Haque [6]. A control group or a non-sepsis group of 70 healthy neonates, matched for gestational age and sex, was also enrolled. The study was conducted over a 2-year duration. Exclusion criteria included neonates with intra-uterine growth restriction (IUGR), infant of diabetic mother (IDM), infants with major congenital anomalies or structural heart diseases, neonates with evidence of perinatal asphyxia with Apgar score < 5 at 5 min, neonates with skin lesions and inability to tolerate adhesive skin leads, neonates with hydrops fetalis, cases on high frequency mechanical ventilation, surgical cases, and infants with septic shock.

The study was conducted in accordance with the Helsinki Declaration. The manuscript was prepared following STROBE guidelines [7].

All the enrolled neonates underwent full history taking and thorough clinical examination.

#### Echocardiography

The first echocardiographic assessment included a full morphologic and hemodynamic assessment of cardiac anatomy and physiology by using a segmental approach [8].

Assessment was performed based on the American Society of Echocardiography guidelines [9] and by using Vivid 7, GE Healthcare, Horten, Norway, to get all the important cardiac views including the standard apical, parasternal, and subcostal views so as to obtain all the quantitative and qualitative parameters.

These parameters included the left ventricle outflow tract area (LVOT) and the velocity time integral (VTI) of aortic blood flow, and both were used to calculate stroke volume (SV) (SV = VTI × LVOT area), cardiac output (CO) (CO = SV × heart rate), cardiac index (CI) (CI = CO/body surface area), systemic vascular resistance (SVR) (SVR = 80 × [Mean Arterial Pressure(MAP)-Central Venous Pressure(CVP)]/CO), and systemic vascular resistance index (SVRI) (SVRI = SVR × BSA) [10, 11].

#### Electrical cardiometry (EC)

Hemodynamic status was assessed using bedside electrical cardiometry in comparison with echocardiography on the 2nd day of the clinical sepsis diagnosis. A portable hand-held EC monitor, routinely applied in our clinical activity (ICON®, Osypka, San Diego-CA, USA), was used according to the manufacturer’s recommendations. Briefly, a high frequency and low amperage current is released through the thorax by two skin electrodes (placed on the forehead and the left thigh); two other electrodes were placed (the left side of the neck and thorax) as far as possible from conventional electrocardiography (ECG) electrodes to receive the signal modified by thoracic impedance. Changes in the impedance are correlated to the ECG captured at the same time [12]. The EC device was connected to the sensor cable, and individual patient data including age, sex, length, weight, non-invasive blood pressure, heart rate (HR), oxygen saturation (SPO2), and hemoglobin level (Hb) were input. The device then continuously displayed the hemodynamic parameters as CO, CI, SV, SVR, and SVRI [13].

EC assessment was performed to all the enrolled neonates in the supine position and during sleep when possible to reduce undesired movements and agitation. Phototherapy, when applied, was discontinued temporarily during this assessment.

#### Study outcomes

- Primary outcomes were to
  - assess the early hemodynamic status in healthy preterm neonates by electrical cardiometry and FoCUS;
  - assess the early hemodynamic status in preterm neonates diagnosed as sepsis by electrical cardiometry and FoCUS

- Secondary outcomes were to
  - evaluate the utility of EC monitoring by comparing the results obtained by its use and FoCUS measurements in septic and healthy preterm neonates.

#### Statistical analysis

Sample size was calculated with a confidence level of = 95% and a power of 80%. Based on a previous study [14], the sensitivity of echocardiography to evaluate cardiac output and cardiac abnormality, as compared with gold standard investigations, is approximated to be 90% among the healthy preterm neonates and the sensitivity of echocardiography expected to be 70% among the septic preterm neonates (due to expected limitations); the least sample size for both of the cases and control groups was (*N* = 70).

Statistical analysis was performed with the Statistical Package for the Social Sciences version 20.0 (SPSS Inc., Chicago, IL, US). Continuous data are presented as mean ± standard deviation or median (interquartile 25–75) for non-normally distributed variables, whereas discrete data are given as absolute values and percentages. Group means of the continuous variables were compared with Student’s *t*-test. Continuous variables were compared between day 2 of sepsis and the last day before discharge using paired samples *t*-test or Wilcoxon’s rank-sum test when appropriate. Categorical variables were compared with chi-squared test or the Fisher exact test when appropriate. Correlations were assessed using Pearson’s correlation test. A *p*-value of < 0.05 was considered statistically significant with a confidence interval of 95%. For agreement, Bland–Altman plot and one sample *t*-test were used (echo and EC) (if significant, then there is fixed bias).

## Results

No statistically significant difference was found between both groups regarding sex, gestational age, postnatal age, and mode of delivery. However, weight and body surface area (BSA) were significantly lower in the sepsis group than the non-sepsis group. Additionally, SPO2/FIO2 ratio was significantly lower in the septic group while serum lactate level was significantly higher in those septic neonates (Table 1).

**Table 1:** Comparison between the two studied groups according to different parameters

	Sepsis (*n* = 70)	Non-sepsis (*n* = 70)	*p*
Sex			
Male	36 (51.4%)	30 (42.9%)	0.310
Gestational age (weeks)			
Mean ± SD	35.2 ± 0.8	35.3 ± 0.9	0.545
Post natal age (days)			
Mean ± SD	8.9 ± 5.2	8.6 ± 5	0.751
5-APGAR (min)			
Median (Min.–max.)	10 (9–10)	10 (9–10)	0.612
Weight (kg)			
Mean ± SD	2.4 ± 0.3	2.6 ± 0.3	0.001^*^
BSA (kg/m^2)^			
Mean ± SD	0.2 ± 0	0.2 ± 0	0.003^*^
Temp (°C)			
Mean ± SD	36.7 ± 0.6	37 ± 0.1	< 0.001^*^
Respiratory rate (c/min)			
Mean ± SD	57 ± 4.2	43 ± 4.9	< 0.001^*^
Heart rate (b/min)			
Mean ± SD	155.6 ± 17	143.9 ± 6.9	< 0.001^*^
SBP (mmHg)			
Mean ± SD	61.7 ± 8.5	65.5 ± 5.8	0.011^*^
DBP (mmHg)			
Mean ± SD	36.8 ± 10.8	42 ± 4.3	0.005^*^
SPO2/FIO2 ratio			
Mean ± SD	367.9 ± 63.59	470.6 ± 49.9	0.000
Serum lactate (mmol/L)			
Mean ± SD	0.72 ± 0.39	0.44 ± 0.29	0.000

*BSA* body surface area, *SBP* systolic blood pressure, *DBP* diastolic blood pressure, *MBP* mean blood pressure, *SPO2* oxygen saturation, *FIO2* fraction of inspired oxygen, *SD* standard deviation, *p*: *p*-value for comparing between the two studied groups

^*^Statistically significant at *p* ≤ 0.05

Cardiac output (CO), stroke volume (SV), and cardiac index (CI), measured by both echocardiography and electrical cardiometry, were significantly higher in the sepsis group than in the non-sepsis group. Meanwhile, systemic vascular resistance (SVR) and systemic vascular resistance index (SVRI) were significantly lower in the sepsis group than in the non-sepsis group, measured with both echocardiography and electrical cardiometry (Table 2).

**Table 2:** Comparison between the two studied groups according to echo and EC

		Sepsis (*n* = 70)	Non-sepsis (*n* = 70)	*p*
SV (ml)	**Echo**			
	Mean ± SD	3.591 ± 0.849	3.107 ± 0.454	< 0.001^*^
	**EC**			
	Mean ± SD	3.577 ± 0.882	3.126 ± 0.451	< 0.001^*^
CO (L/min)	**Echo**			
	Mean ± SD	0.554 ± 0.145	0.441 ± 0.055	< 0.001^*^
	**EC**			
	Mean ± SD	0.553 ± 0.151	0.443 ± 0.055	< 0.001^*^
CI (L/min/m^2^)	**Echo**			
	Mean ± SD	3.301 ± 0.891	2.482 ± 0.251	< 0.001^*^
	**EC**			
	Mean ± SD	3.297 ± 0.927	2.489 ± 0.252	< 0.001^*^
SVR (dyn-s/cm^5)^	**Echo**			
	Mean ± SD	6548.7 ± 2289	8547.5 ± 1019.8	< 0.001^*^
	**EC**			
	Mean ± SD	6672.9 ± 2577.6	8533.4 ± 1026.3	< 0.001^*^
SVRI(dyn-s/cm^5^)/m^2^	**Echo**			
	Mean ± SD	1102.5 ± 370.7	1523.8 ± 232.3	< 0.001^*^
	**EC**			
	Mean ± SD	1122.6 ± 433	1519.2 ± 220.2	< 0.001^*^

*SV* stoke volume, *CO* cardiac output, *CI* cardiac index, *SVR* systemic vascular resistance, *SVRI* systemic vascular resistance index, *SD* standard deviation, *p*: *p*-value for comparing between the two studied groups

^*^Statistically significant at *p* ≤ 0.05

**Fig. 1:**
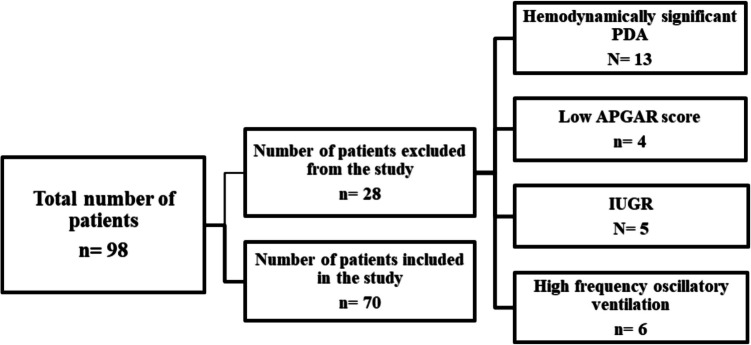
Correlation between echo and EC parameters in each sepsis group (*n* = 70). SV (ml) correlation (*r*: 0.935; *p* < 0.001*). SV, stoke volume. *r*_s_: Spearman coefficient; *Statistically significant at *p* ≤ 0.05

**Fig. 2:**
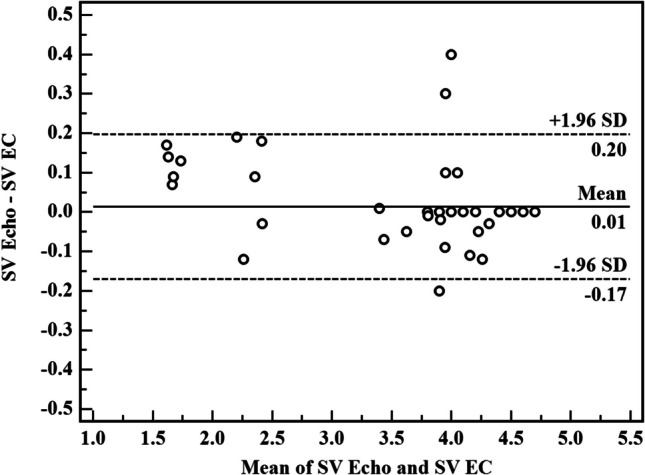
Correlation between echo and EC parameters in each sepsis group (*n* = 70). CO (L/min) correlation (*r*: 0.995; *p* < 0.001*). CO, cardiac output. *r*_s_: Spearman coefficient; *Statistically significant at *p* ≤ 0.05

**Fig. 3:**
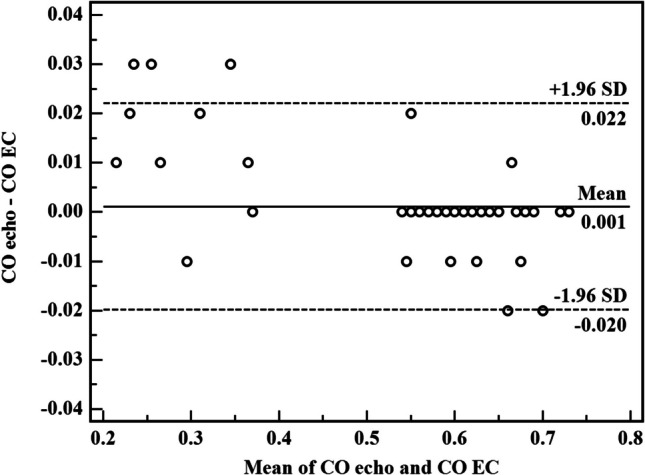
Correlation between echo and EC parameters in each sepsis group (*n* = 70). CI (L/min/m.^2^) correlation (*r*: 0.994; *p* < 0.001*). CI, cardiac index. *r*_s_: Spearman coefficient; *Statistically significant at *p* ≤ 0.05

**Fig. 4:**
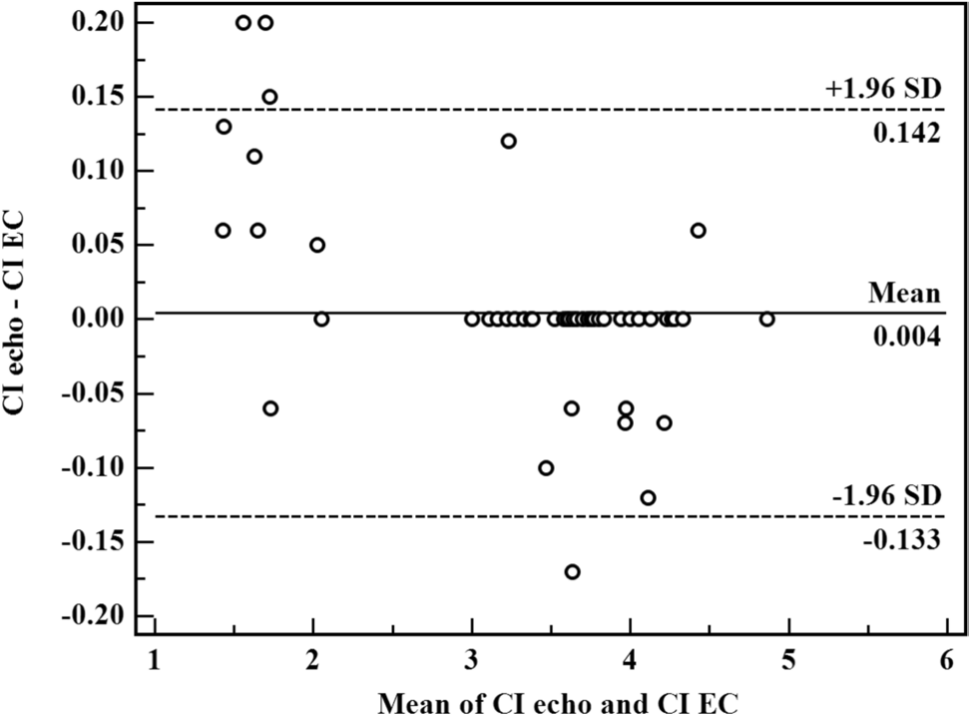
Correlation between echo and EC parameters in each sepsis group (*n* = 70). SVR (dyn-s/cm.^5^) correlation (*r*: 0.979; *p* < 0.001*). SVR, systemic vascular resistance. *r*_s_: Spearman coefficient; *Statistically significant at *p* ≤ 0.05

**Fig. 5:**
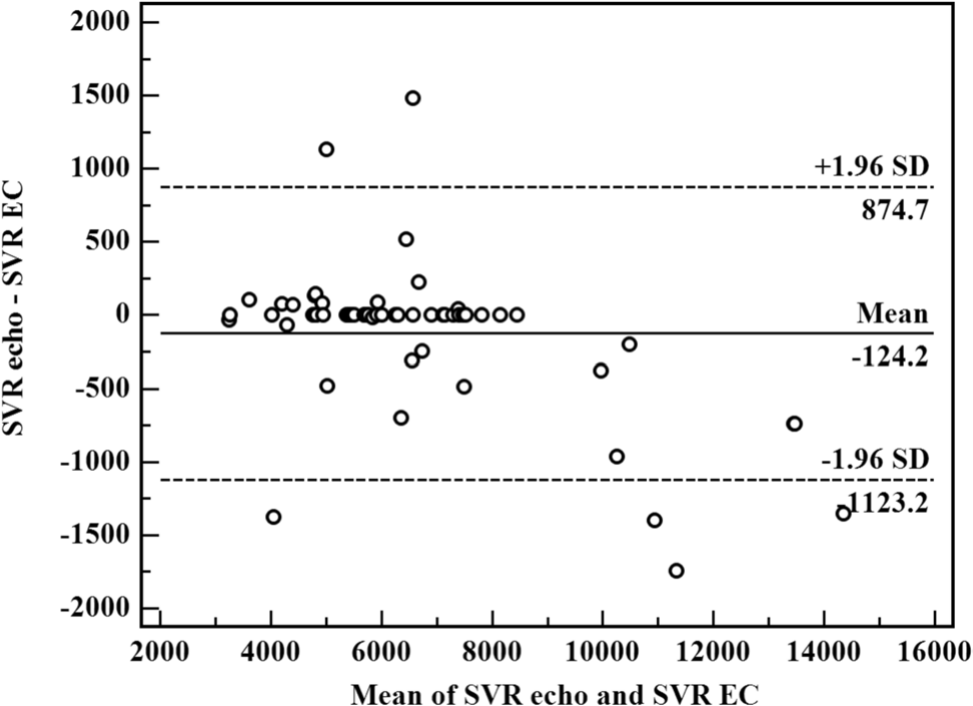
Correlation between echo and EC parameters in each sepsis group (*n* = 70). SVRI (dyn-s/cm^5^)/m.^2^ correlation (*r*: 0.986; *p* < 0.001*). SVRI, systemic vascular resistance index. *r*_s_: Spearman coefficient; *Statistically significant at *p* ≤ 0.05

In the sepsis group, measurements obtained by electrical cardiometry (EC) including stroke volume (SV), cardiac output (CO), cardiac index (CI), systemic vascular resistance (SVR), and systemic vascular resistance index (SVRI) showed a statistically significant positive correlation with the corresponding parameters assessed by FoCUS, as shown in Figs. 1, 2, 3, 4, and 5.

Agreements were calculated by the Bland–Altman analysis (inserted as an online supplement).

## Discussion

Many infants with sepsis develop cardiovascular instability; preterm infants are particularly vulnerable due to the unique features of their cardiovascular function and reserve [15].

Characterization of sepsis-related cardiovascular dysfunction has been traditionally based on the clinical patterns identified by bedside physical examination, typically dichotomized as warm and cold shock physiology [16].

The hemodynamic status of sick newborn infants is often assessed by clinical variables such as heart rate, blood pressure, and capillary refill time, which have been demonstrated to be misleading in their accuracy [17].

Early recognition of cardiovascular compromise in sick infants enables the physician to take timely therapeutic decisions and monitor response to treatment more objectively [18].

Bedside echocardiography is an ideal method for monitoring hemodynamic assessment in neonates. It can assess cardiac function, preload, afterload, and cardiac output [19].

Electrical cardiometry (EC) has been proposed as a safe, accurate, and reproducible technique for hemodynamic measurement in children and infants [20].

EC measures alteration in thoracic resistance or impedance using four skin electrodes. EC is able to isolate the changes in impedance created by the circulation, partly caused by the change in orientation of the erythrocytes during the cardiac cycle [21].

In this study, our aims were to assess the early hemodynamic status and cardiac performance in healthy preterm neonates (non-sepsis group) and in the sepsis group by electrical cardiometry in comparison to echocardiography.

In the current study, there was no statistically significant difference between both groups regarding sex, weight, gestational age, postnatal age, mode of delivery, SPO2/FIO2 ratio, and serum lactate level.

Mean blood pressure is commonly used in the NICUs as a simple, continuous, bedside measure of adequate perfusion in a neonate. This facilitates monitoring hemodynamics and response to treatment. However, it is limited by the lack of robust normative values in both term and preterm babies [22].

The heart rate was higher in the sepsis group. This could be explained by sympathetic dysfunction, as both overactivation and downregulation have been described in sepsis. Activation of the adrenergic system in sepsis, which is important for a physiological response to infection, if excessive, may result in overproduction of catecholamines that leads to tachycardia [23].

Stroke volume and cardiac output values of the healthy preterm neonates enrolled in this study came in agreement with Boet et al. [24], while the mean values of SV, CO, and CI in the sepsis group (3.6 ± 0.8 ml, 0.6 ± 0.1 L/min, 3.3 ± 0.9 L/min/m^2^ respectively measured with FoCUS, and 3.6 ± 0.9 ml, 0.6 ± 0.2 L/min, 3.3 ± 0.9 L/min/m^2^ by EC) were significantly higher compared to the same parameters levels obtained from the neonates of the non-sepsis group (3.1 ± 0.5 ml, 0.4 ± 0.1 L/min, 2.5 ± 0.3 L/min/m^2^ respectively measured with echo and 3.1 ± 0.5 ml, 0.4 ± 0.1 L/min, 2.5 ± 0.3 L/min/m^2^ by EC).

In contrast, the mean values of SVR and SVRI were significantly lower in the sepsis group (6548.7 ± 2289 dyne-s/cm^5^ and 1102.5 ± 370.7 (dyne-s/cm^5^)/m^2^ by FoCUS and 6651.6 ± 2590.3 dyne-s/cm^5^ and 1122.6 ± 433 (dyne-s/cm^5^)/m^2^ by EC) than those measured in the non-sepsis group (8547.5 ± 1019.8 dyne-s/cm^5^ and 1523.8 ± 232.3 (dyne-s/cm^5^)/m^2^ by FoCUS and 8533.4 ± 1026.3 dyne-s/cm^5^ and 1519.2 ± 220.2 (dyne-s/cm^5^)/m^2^ by EC).

These findings can be explained by the different anatomical structure of the premature heart. The immature heart has a lower mass, fewer and less organized myofibrils, fewer mitochondria, fewer L-type calcium channels, and shallower T-tubules, resulting in decreased the ability to facilitate the release of calcium from sarcoplasmic reticulum, higher overall collagen content as well as a higher ratio of collagen rigidity-increasing type I to elasticity-increasing type, and less adrenergic innervation and adrenoreceptor density [25].

Functionally, these anatomic differences are translated into lower functional reserve in response to altered loading conditions and stresses, lower diastolic performance, less ability to increase stroke volume in response to increases in preload, and a greater tendency for systolic dysfunction and lower stroke volume in face of acute increases in afterload [15, 26].

While individual variations may occur, the predominant physiological response to sepsis in the vascular system also appears to be developmentally regulated. In adult patients, predominant physiology is known to be that of warm shock, whereas in children, sepsis tends to produce primarily cold shock physiology. Physiological studies in preterm infants, similar to adults, have demonstrated warm shock physiology to be the predominant phenotype. This is postulated to be due to an impaired ability to regulate vascular tone during shock, in part driven by an inherent imbalance of the autonomic nervous system characterized by a relatively higher parasympathetic drive. Relative adrenal insufficiency, by decreasing SVR, may also be a contributing factor in preterm infants [15].

The Pearson coefficient test was used to evaluate the correlation between the EC measurements and the echocardiogram measurements. SV, CO, CI, SVR, and SVRI measurements by EC showed significant positive correlation with FoCUS measurements among sepsis group cases.

Bland–Altman plots were made to evaluate the agreement of SV, CO, and CI readings by echo and EC. Bias was defined as the mean difference between the EC and echo measurements. In sepsis group, the mean bias of SV reading was 0.02 ± 0.098 (limits of agreement − 0.17–0.21). The mean bias of CO reading was 0.000 ± 0.004 with (limit of agreement of − 0.008–0.008). The mean bias of CI reading was 0.000 ± 0.024 with (limit of agreement of − 0.048–0.047). These differences show an accepted degree of agreement between EC and echocardiogram that are nearly equivalent in evaluation of the beforementioned hemodynamic measurement in preterm neonates diagnosed with sepsis owing to the tiny bias and narrow limits.

The current study had some limitations; it was a single-center study, sample size was relatively small, preterm neonates with septic shock were excluded from the study as many neonates could be presented with different types of septic shock with variable hemodynamics which require a separate study. Furthermore, cases on HFOV were not included as many authors have suggested reduced EC accuracy under HFOV, lack of studies about the reproducibility of the EC in neonates, the time points of evaluation can be variable within first 24 h of age and on the 3rd day, and neonates with different respiratory disorders like RDS were not included.

## Conclusions

Neonatal sepsis is a unique hemodynamic state, and neonates with sepsis showed high cardiac output as demonstrated by echocardiography and electrical cardiometry. There was a strong correlation between cardiac output measurements obtained by electrical cardiometry and FoCUS. Electrical cardiometry can be recommended as a useful tool for continuous hemodynamics assessment in the preterm neonates with sepsis. Both FoCUS and EC can be used for hemodynamics assessment in NICUs.

## Supplementary Information

Below is the link to the electronic supplementary material.

Supplementary file1 (DOCX 207 KB)

## Data Availability

No datasets were generated or analysed during the current study.

## Abbreviations

ASD: Atrial septal defect
CI: Cardiac index
CO: Cardiac output
EC: Electrical cardiometry
ECG: Electrocardiogram
FoCUS: Focused cardiac ultrasound
GA: Gestational age
Hb: Hemoglobin
HR: Heart rate
I/T: Immature/total ratio
IDM: Infant of diabetic mother
IUGR: Intra-uterine growth restriction
MBP: Mean blood pressure
NICU: Neonatal intensive care unit
PDA: Patent ductus arteriosus
PFO: Patent foramen ovale
PLT: Platelets count
SV: Stroke volume
SVR: Systemic vascular resistance
SVRI: Systemic vascular resistance index
TLC: Total leukocytes count

## References

[1] Simonsen KA, Anderson-Berry AL, Delair SF, Davies HD (2014) Early-onset neonatal sepsis. Clin Microbiol Rev 27(1):21–47. 10.1128/CMR.00031-13 24396135 10.1128/CMR.00031-13 PMC3910904

[2] Voller SMB, Myers PJ (2016) Neonatal sepsis. Clin Pediatr Emergency Med 17(2):129–133. 10.1016/j.cpem.2016.03.006

[3] Duignan SM, Lakshminrusimha S, Armstrong K, de Boode WP, El-Khuffash A, Franklin O, Molloy EJ, de Boode WP, Plötz FB, Strunk T, Degtyareva M, Küster H, Giannoni E, Bliss JM, Taal HR, Klingenberg C, Naver L, van den Hoogen A (2023) Neonatal sepsis and cardiovascular dysfunction I: mechanisms and pathophysiology. Pediatr Res. 10.1038/s41390-023-02926-2 38044334 10.1038/s41390-023-02926-2

[4] Gatelli IF, Vitelli O, Fossati M, De Rienzo F, Chiesa G, Martinelli S (2022) Neonatal septic shock and hemodynamic monitoring in preterm neonates in an NICU: added value of electrical cardiometry in real-time tailoring of management and therapeutic strategies. Am J Perinatol 39(13):1401–1404. 10.1055/s-0041-1726123 33723835 10.1055/s-0041-1726123

[5] Ballard JL, Khoury JC, Wedig KL, Wang L, Eilers-Walsman BL, Lipp R (1991) New Ballard score, expanded to include extremely premature infants. J Pediatr 119(3):417–423. 10.1016/s0022-3476(05)82056-6 1880657 10.1016/s0022-3476(05)82056-6

[6] Haque KN (2005) Definitions of bloodstream infection in the newborn. Pediatr Crit Care Med : a J Soc Crit Care Med World Federation Pediatr Intensive Critical Care Soc 6(3 Suppl):S45–S49. 10.1097/01.PCC.0000161946.73305.0A 10.1097/01.PCC.0000161946.73305.0A 15857558

[7] Vandenbroucke JP, von Elm E, Altman DG, Gøtzsche PC, Mulrow CD, Pocock SJ, Poole C, Schlesselman JJ, Egger M, & STROBE Initiative (2007). Strengthening the Reporting of Observational Studies in Epidemiology (STROBE): explanation and elaboration. Epidemiology (Cambridge, Mass.) 18(6):805–835. 10.1097/EDE.0b013e3181577511 10.1097/EDE.0b013e3181577511 18049195

[8] Lai WW, Geva T, Shirali GS, Frommelt PC, Humes RA, Brook MM, Pignatelli RH, Rychik J. Task Force of the Pediatric Council of the American Society of Echocardiography, & Pediatric Council of the American Society of Echocardiography 2006 Guidelines and standards for performance of a pediatric echocardiogram: a report from the Task Force of the Pediatric Council of the American Society of Echocardiography. J Am SocEchocardiogr Off Publ Am Soc Echocardiogr 19(12):1413–1430 10.1016/j.echo.2006.09.001 10.1016/j.echo.2006.09.001 17138024

[9] Mertens L, Seri I, Marek J, Arlettaz R, Barker P, McNamara P, Moon-Grady AJ, Coon PD, Noori S, Simpson J, Lai WW, Writing Group of the American Society of Echocardiography, European Association of Echocardiography, & Association for European Pediatric Cardiologists (2011). Targeted neonatal echocardiography in the neonatal intensive care unit: practice guidelines and recommendations for training. Writing Group of the American Society of Echocardiography (ASE) in collaboration with the European Association of Echocardiography (EAE) and the Association for European Pediatric Cardiologists (AEPC). J Am Soc Echocardiogr : Off Publ Am Soc Echocardiogr 24(10):1057–1078. 10.1016/j.echo.2011.07.014 10.1016/j.echo.2011.07.014 21933743

[10] Stamler JS, Loh E, Roddy MA, Currie KE, Creager MA (1994) Nitric oxide regulates basal systemic and pulmonary vascular resistance in healthy humans. Circulation 89(5):2035–2040. 10.1161/01.cir.89.5.2035 7514109 10.1161/01.cir.89.5.2035

[11] Yoon SJ, Han JH, Cho KH, Park J, Lee SM, Park MS (2022) Tools for assessing lung fluid in neonates with respiratory distress. BMC Pediatr 22(1):1–7. 10.1186/s12887-022-03361-8 35725416 10.1186/s12887-022-03361-8 PMC9208096

[12] Bisceglie V, Loi B, Vitelli O, Proto A, Ferrari ME, Vivalda L, Nardo MD, Martinelli S, De Luca D (2024) Neonatal reference values and nomograms of systemic vascular resistances estimated with electrical cardiometry. J Perinatol 45(3):334–341. 10.1038/s41372-024-02115-x 39289555 10.1038/s41372-024-02115-x

[13] O'Neill R, Dempsey EM, Garvey AA, Schwarz CE (2021) Non-invasive cardiac output monitoring in neonates. Front Pediatr 8:614585. 10.3389/fped.2020.614585.10.3389/fped.2020.614585 PMC7880199 33585366

[14] Singh Y (2017) Echocardiographic evaluation of hemodynamics in neonates and children. Front Pediatr 5:201. 10.3389/fped.2017.00201 28966921 10.3389/fped.2017.00201 PMC5605552

[15] Kharrat A, Jain A (2022) Hemodynamic dysfunction in neonatal sepsis. Pediatr Res 91(2):413–424. 10.1038/s41390-021-01855-2 34819654 10.1038/s41390-021-01855-2

[16] Weiss SL, Peters MJ, Alhazzani W, Agus MSD, Flori HR, Inwald DP, Nadel S, Schlapbach LJ, Tasker RC, Argent AC, Brierley J, Carcillo J, Carrol ED, Carroll CL, Cheifetz IM, Choong K, Cies JJ, Cruz AT, De Luca D, Deep A, … Tissieres, P. (2020). Surviving sepsis campaign international guidelines for the management of septic shock and sepsis-associated organ dysfunction in children. Pediatric critical care medicine: a journal of the Society of Critical Care Medicine and the World Federation of Pediatric Intensive and Critical Care Societies 21(2):e52–e106. 10.1097/PCC.0000000000002198 10.1097/PCC.0000000000002198 32032273

[17] Deshpande S, Suryawanshi P. Chaudhary N, Maheshwari R (2017) Cardiac output in late onset neonatal sepsis. J Clin of Diagn Res. 11(11):SC25-SC28. 10.7860/JCDR/2017/30312/10871

[18] El-Khuffash AF, McNamara PJ (2011) Neonatologist-performed functional echocardiography in the neonatal intensive care unit. Semin Fetal Neonatal Med 16(1):50–60. 10.1016/j.siny.2010.05.001 20646976 10.1016/j.siny.2010.05.001

[19] Shokr AAES, Tomerak RH, Agha H, Elkaffas R, Ali S (2023) Echocardiography-directed management of hemodynamically unstable neonates in tertiary care hospitals. Egypt Pediatric Association Gaz 71:10. 10.1186/s43054-023-00157-y

[20] Hsu KH, Wu TW, Wang YC, Lim WH, Lee CC, Lien R (2016) Hemodynamic reference for neonates of different age and weight: a pilot study with electrical cardiometry. J Perinatol: official journal of the California Perinatal Association 36(6):481–485. 10.1038/jp.2016 10.1038/jp.2016.2 26890553

[21] Sanders M, Servaas S, Slagt C (2020) Accuracy and precision of non-invasive cardiac output monitoring by electrical cardiometry: a systematic review and meta-analysis. J Clin Monit Comput 34(3):433–460. 10.1007/s10877-019-00330-y 31175501 10.1007/s10877-019-00330-y PMC7205855

[22] El-Khuffash A, McNamara PJ (2017) Hemodynamic assessment and monitoring of premature infants. Clin Perinatol 44(2):377–393. 10.1016/j.clp.2017.02.001 28477667 10.1016/j.clp.2017.02.001

[23] Dünser MW, Hasibeder WR (2009) Sympathetic overstimulation during critical illness: adverse effects of adrenergic stress. J Intensive Care Med 24(5):293–316. 10.1177/0885066609340519 19703817 10.1177/0885066609340519

[24] Boet A, Jourdain G, Demontoux S, De Luca D (2016) Stroke volume and cardiac output evaluation by electrical cardiometry: accuracy and reference nomograms in hemodynamically stable preterm neonates. J Perinatol 36(9):748–752. 10.1038/jp.2016.65 27101386 10.1038/jp.2016.65

[25] Kane C, Couch L, Terracciano CM (2015) Excitation-contraction coupling of human induced pluripotent stem cell-derived cardiomyocytes. Front Cell Dev Biol 3:59. 10.3389/fcell.2015.00059 26484342 10.3389/fcell.2015.00059 PMC4586503

[26] Bensley JG, Moore L, De Matteo R, Harding R, Black MJ (2018) Impact of preterm birth on the developing myocardium of the neonate. Pediatr Res 83(4):880–888. 10.1038/pr.2017.324 29278645 10.1038/pr.2017.324

