# Supplementary material for: FoCUS and non-invasive hemodynamics monitoring in neonatal sepsis
Source: Eur J Pediatr. 2025 Jun 30;184(7):456. doi: 10.1007/s00431-025-06283-6 (PMC12206674; doi:10.1007/s00431-025-06283-6)
Supplement: Supplementary file 1 — Supplementary file1 (DOCX 207 KB) [file 431_2025_6283_MOESM1_ESM.docx]

**Figures (1, 2, 3, 4, and 5): Bland Altman for agreement between Echo and EC in Sepsis group (n = 70):**

| **Bland Altman for agreement between Echo and EC in Sepsis group (n = 70)** | |
| --- | --- |
| **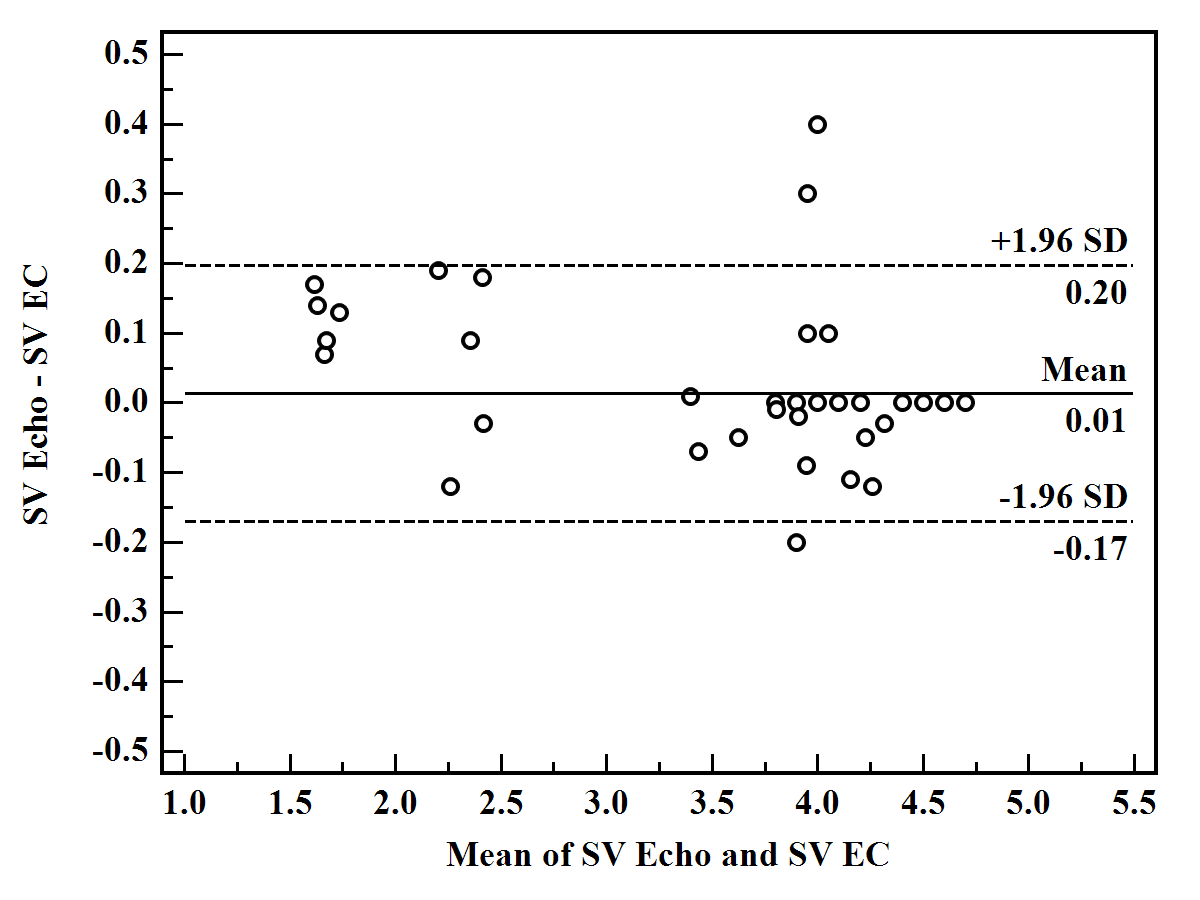** | 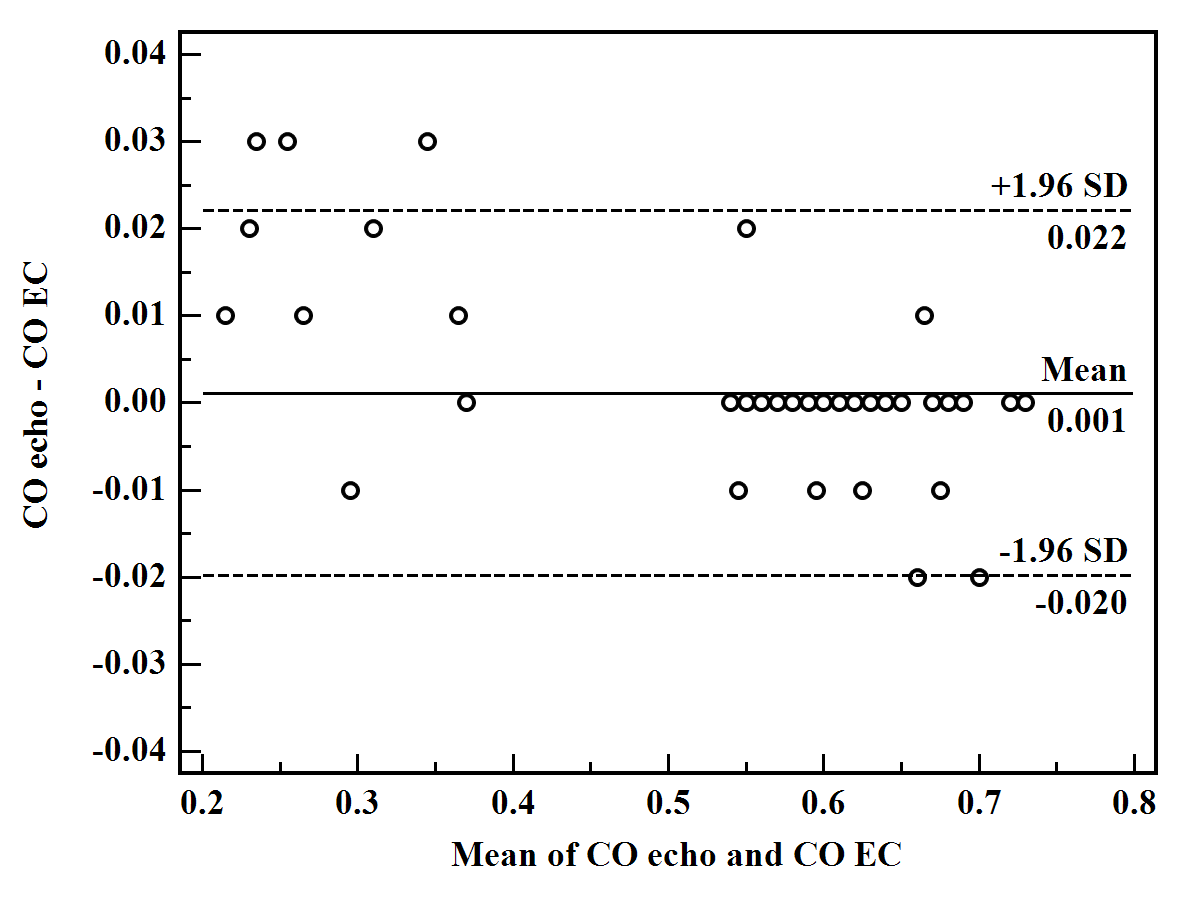 |
| **Figure (1): Bland Altman for SV** | **Figure (2): Bland Altman for CO** |
| **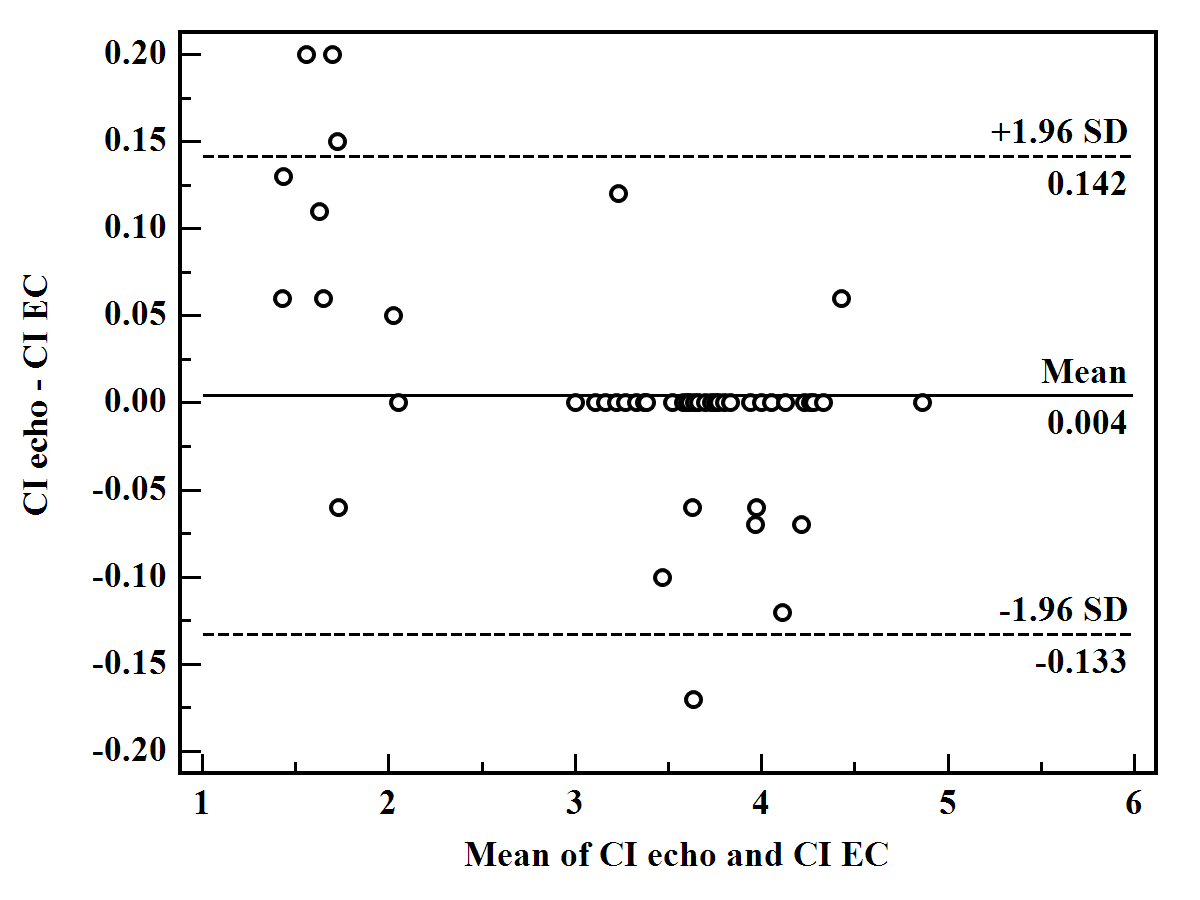** | 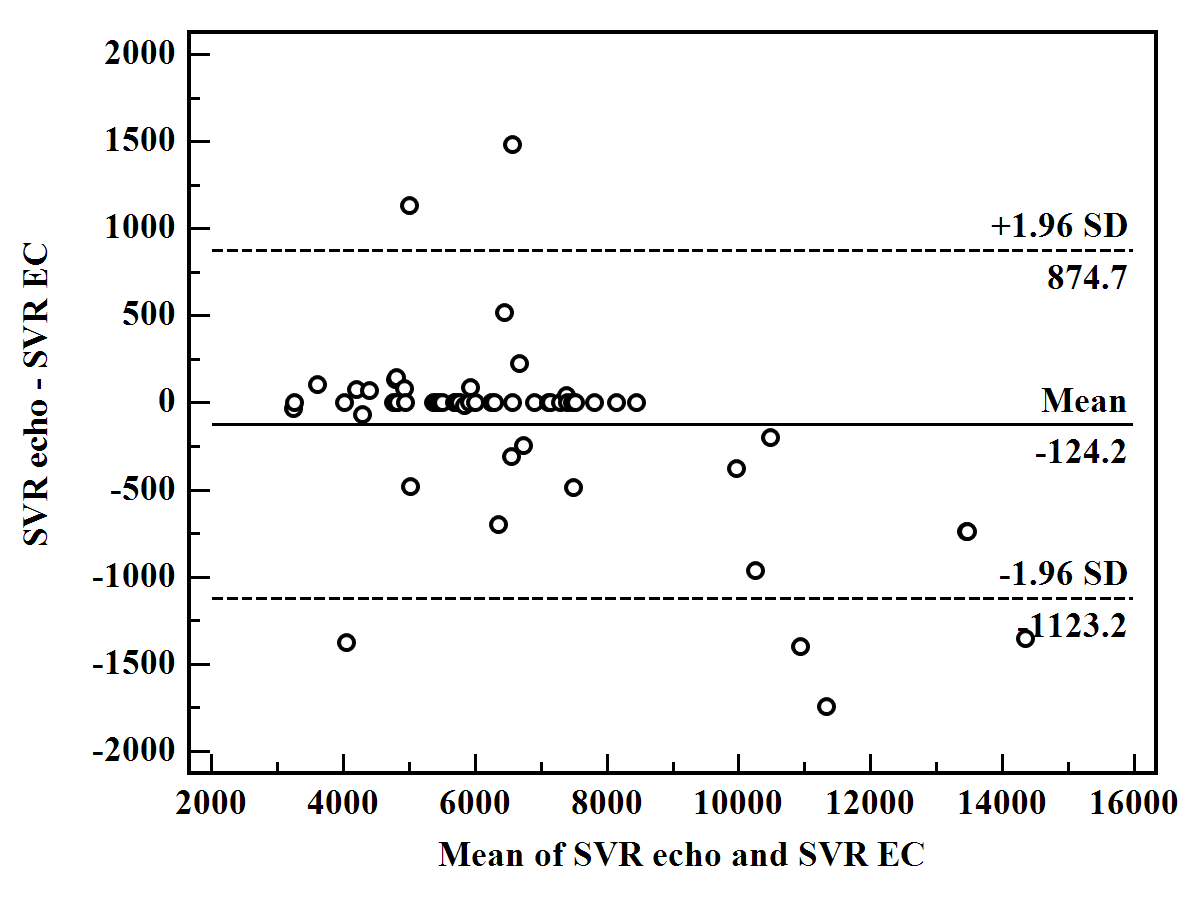 |
| **Figure (3): Bland Altman for CI** | **Figure (4): Bland Altman for SVR** |
| **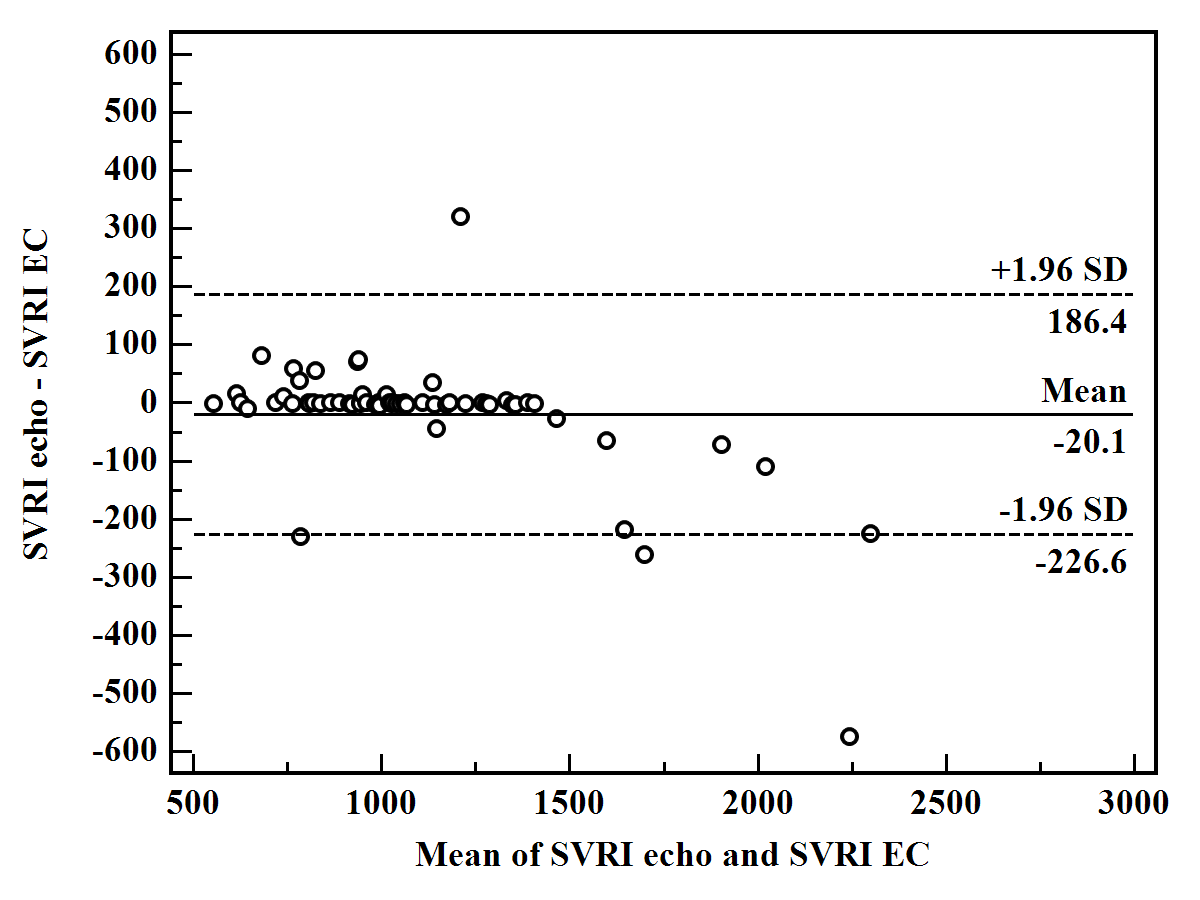** | |
| **Figure (5): Bland Altman for SVRI** | |
